# Medical Association of Georgia

**Published:** 1897-05

**Authors:** 


### SOCIETY REPORTS.

### MEDICAL ASSOCIATION OF GEORGIA.

The Forty-eighth Annual Meeting of the Association was held at Macon, Ga., April 21, 22, and 23, 1897.

The Association convened in the Academy of Music, and was called to order at 10 A. M. by the President, Dr. Geo. H. Noble, of Atlanta.

Addresses of welcome were delivered by Dr. W. F. Holt and Hon. N. E. Harris, of Macon, the response to which was made by Dr. Frank M. Ridley, of LaGrange.

The Society elected one hundred and one new members.

After the presentation of the Report of the Committee of Arrangements by Dr. Howard J. Williams, of Macon, and the report of the Committee on Program by Dr. Louis H. Jones, of Atlanta, the reading of papers was proceeded with.

The first paper read was by Dr. M. A. Clark, of Macon, entitled "Entero-Colitis of Infancy." (To be published in this JOURNAL.)

Dr. K. P. Moore, of Macon, reported some very interesting and unusual cases in abdominal and gynecological work.

Dr. Henry R. Slack, of LaGrange, read a paper with this caption, "Blue Pyoktanin in the Treatment of Inoperable Malignant Growths," and reported five cases.

The author at first referred to contributions on this subject by Professor Von Mosetig and Willy Meyer. The author speaks of its advantages as follows:

1. Its analgesic effects are marked, as patients soon rest easily without the aid of morphine.

2. The improvement of the function of the part involved. A man who could hardly speak so as to be understood talked without difficulty after the third injection.

3. The improvement in general health which has taken place in all five of the cases.

4. The element of hope that is added to the life of suffering men, brightening the remainder of their sojourn.

While the author does not claim to have cured his patients, still he has relieved their pains and rendered them less burdensome to themselves and their friends. He agrees with Dr. Meyer in Von Mosetig's conclusions, that it has been proved by practice that parenchymatous injections of inoperable malignant growths with pyoktanin can produce disappearance of malignant tissue, though in exceptional cases, and can heal neoplastic ulcerations. Pyoktanin, when properly used, is certainly a palliative treatment for cancer that deserves an honest, hopeful trial, for by its use many have been relieved and some cured.

Dr. J. B. Morgan, of Augusta, read a paper entitled "The Treatment of Cutaneous Cancers."

In discussing the treatment of skin cancers, it is well to understand at the beginning that the author does not intend to define the different varieties of cancer, or to discuss the different theories as to their etiology. A fact of paramount importance in the successful treatment of cancers of the skin is that everything depends upon early and thorough removal of all diseased tissue. The general consensus of opinion now is that all cancers can be cured, if thoroughly removed before the epithelial cells have infiltrated the connective tissue and neighboring glands; for it is generally conceded that it is the proliferation and infiltration of the epithelial cells which cause cancer to invade other tissues and organs and become disseminated through the system. This being the case, the only treatment that can offer anything like a cure is the one which has for its object the early and complete removal, or destruction, ot all the morbid epithelia. In cancer, where we find no limiting membrane, no sharply defined guide as to the extent of the infiltration beyond the tumor mass, other local means than the knife may be more properly indicated for its successful removal. The object of the author's paper was to emphasize this point. He is a strong believer in the use of the knife in suitable cases, and frequently employs it.

In the last four years he had treated fourteen cases of skin cancers with caustic, and in not a single instance has he seen the slightest indication of recurrence. Five of the cases were from three to four years old; three of them were from two to three years old, and the other six were treated within the last two years. One of the number died from pneumonia three years after being operated upon; the others are all living and in good health. Caustic potash quickly liquefies tissue, and at one sitting a very large tumor can be destroyed. Chloride of zinc as a caustic can be used in stick form, in solution, or in a paste. It causes more pain than either caustic potash or arsenious acid. Bougard's paste is one of the best means we possess in the treatment of small cutaneous cancers. To apply these caustics successfully demands a certain amount of experience, but which any one can acquire after applying them two or three times. The greatest cause of failure in their use is the neglect to treat the case energetically until thoroughly satisfied that all diseased tissue has been removed.

Dr. S. Rumble, of Goggansville, followed with a paper entitled "Puerperal Eclampsia, with Report of Cases." The author presented the following conclusions:

1. That puerperal eclampsia is a disease the etiology of which is as yet *sub judice*.

2. That it often develops after the uterus is emptied of the product of conception.

3. That morphia is the most positive and prompt remedy which can be used to arrest the eclamptic seizure. It obtunds nervous sensibility and arrests muscular spasm. It counteracts irritation of the nerve centers, whether the irritation be due to toxemia or is of a reflex character.

4. That veratrum is not only an unreliable remedy, but one fraught with danger in this disease.

5. That chloroform and chloral hydrate are excellent adjuvants to morphia in arresting the spasms and maintaining the equilibrium of the nervous centers.

6. That cerebral congestion is a result, and not the cause, of the convulsion. Hence the author does not favor blood-letting.

7. We can do a great deal in lessening the frequency, if not the ratio of mortality in this disease by timely and proper prophylactic treatment.

Dr. M. B. Hutchins, of Atlanta, reported "Two Cases of Malignant Tumor Affecting the Breast, and Their Treatment," and made some remarks on malignant growths in general. (Will appear in *International Journal of Surgery;* also this JOURNAL.)

Dr. J. S. Todd, of Atlanta, contributed a paper entitled, "Cause and Prevention of Tuberculosis. Is it a Curable Disease?" (To be published in this JOURNAL.)

Dr. J. C. Olmsted, of Atlanta, followed with a paper on "Expert Testimony in Criminal Cases Involving the Plea of Insanity."

Dr. Olmsted called attention to the present unfortunate methods of obtaining medical expert testimony, and advocated the enactment of suitable legislation which could obviate these evils.

Dr. V. D. Lockhart, of Maysville, reported an interesting and instructive case of cystocele complicating labor.

Dr. E. H. Richardson, of Atlanta, read a paper entitled "Atypical Fevers."

Dr. W. L. Champion, of Atlanta, followed with a contribution entitled "Meddlesome Instrumentation in Urethral Diseases." (To be published in this JOURNAL.)

Dr. E. R. Anthony, of Griffin, contributed a paper on "The Relation of the State Board of Medical Examiners to the Profession of the State." (Published in this issue.)

Dr. Louis H. Jones, of Atlanta, read a paper entitled "Physical Signs in Diseases of the Lungs." Dr. Jones took the position that the crepitant or so-called vesicular râle was a sound always produced by the pleuræ, that the generally accepted view that the râle was caused by the separation of aglutinated cell walls, or the bubbling of air through liquid in the cells and finer bronchi, was not in accord with the physical laws governing respiration, and that it must be explained in some other way. He maintained crepitation could frequently be heard in pneumonia, after complete consolidation had occurred, and that it was absent in those rare cases in which the lesion in the lung tissue was not accompanied by pleurisy.

Dr. A. W. Stirling, of Atlanta, followed with a contribution entitled "Remarks on Certain Ocular Disturbances of General Interest." The author confined himself to one or two of those ocular lesions which are likely to be of general interest. Of conjunctival affections he mentioned one-sided hyperemia, which he said should always suggest a foreign body in the conjunctival sac or on the cornea. Few of the minor operations in surgery would better repay the practitioner than a dexterous manipulation of the eye in such cases, and one should not be hasty in excluding a foreign body.

Passing to the cornea, the speaker said that our chief interest lies with ulcers and with foreign bodies. The latter were often difficult to find, and when found may be deeply embedded in the corneal tissue, and not always quite easy of removal. Even after the simplest abrasion by the foreign body, or by the instrument used in its removal, the protecting epithelium was destroyed, and not infrequently germs thereby gain access to the corneal tissue, and severe ulceration, with or without hypopyon results. Strict antisepsis was therefore advisable, frequently also a bandage for twenty-four hours, and sometimes atropin. Corneal abscesses and ulcers had a very varied etiology, and were often dependent upon general ill health. They usually demanded shade, atropin, and antiseptic applications, but when near the periphery eserine might replace atropin in the absence of iritis. Should the ulcer perforate, there was then less likelihood of prolapse of the iris into the wound. Some of the severest forms of ulcer might be rapidly cut short by certain destructive agents, notably the electric cautery, the sharp spoon, strong solutions of perchloride of mercury, mercury in glycerin or alcohol, and frequently very prompt treatment in them also is required to save the whole eye from destruction.

While cataract is looked upon as usually a purely local disturbance, except in the rare cases in which it is associated with certain diseases, as diabetes, there is one form of opacity in the lens which is believed to have important relationships to conditions of general health. This is called zonular or lamellar, and sometimes congenital cataract, and is by far the most frequent type of lenticular opacity met with in children. It affects a varying amount of the lens, and the opacity produced by it may be so slight as to interfere but little with vision, or so great as to disqualify its owner for ordinary work.

The author then passed on to the consideration of iritis, saying that it was a fairly common disease, and one which, on account of circumstances, has frequently to be treated in great measure by the non-specialist. The likelihood of an attack proving destructive to vision depends to a very great extent upon its early recognition and prompt and energetic treatment.

Dr. Samuel Lloyd, of New York, gave an excellent demonstration of the Roentgen rays. He showed skiagraphs of a case of knock-knee, of congenital disappearance of the fingers and toes, and a case of scissor-legs, upon which he operated some weeks ago. The latter patient was a young girl whose thigh bones were crossed, thus preventing her from using the limbs. About nine weeks ago she was taken to the Post-Graduate Hospital of New York, where Dr. Lloyd performed the operation of osteotomy. Both thigh bones were fractured and placed in position for a normal re-union. The bones were subjected to a ten-minute exposure of the ray, and by the use of the fluoroscope Dr. Lloyd was able to announce a perfect union of the bones. He related other experiments which were equally successful, which were made with fractured bones of the knee and elbow and with diseased bones of the hand. Dr. Lloyd also showed skiagraphs of a case of fracture of the forearm and one of the osteoma of the clavicle.

Dr. R. M. Harbin, of Rome, read a paper entitled "Rapid Dilatation of the Uterus a Conservative Operation."

His attention was recently called to an article in the *British Medical Journal*, by Dr. Fournel, condemning rapid dilatation of the uterus as not being a conservative operation. So far as his observation goes, the dangers from the operation were very slight where proper precautions have been taken. Divulsion of the uterus offers more relief in selected cases than the more complicated and dangerous operations, and should be done before advising the latter. The main indications for the operation are narrowness of the os and cervix of the uterus, flexions with dysmenorrhea and sterility, and the reflex symptoms arising from same. Personal observation leads the author to believe that many cases of obstinate nausea and vomiting of pregnancy could be prevented by rapid dilatation shortly before an expected conception, as the practitioner often had to perform abortion in cases that conceived very soon afterwards, rendering the operation again necessary. The cause of dysmenorrhea and sterility, in a majority of cases, was mechanical, and dilatation is usually followed by a relief of the symptoms, if not a cure of the sterility. The contraindications are pyosalpinx and acute peritonitis. In an aggravated case of chronic pelvic peritonitis, rapid dilatation can be done with safety after a few weeks of general and local treatment. The operation should be done about a week before menstruation, and the technique was simple.

Dr. Wm. S. Goldsmith, of Atlanta, made some remarks on "Fistula in Ano." (To be published in this JOURNAL.)

Dr. Hunter McGuire, of Richmond, Va., read a paper (by invitation), entitled "Remarks on Appendicitis," with a report of twenty-six cases operated on during the past twelve months. The author considered at the outset the importance of operating for appendicitis during an interval between attacks. All of the nineteen cases of chronic or relapsing appendicitis embraced in the report recovered. In looking over his record for previous years he finds that he has operated on thirty-three other cases, with only one fatal result, thus making a total of fifty-two consecutive operations for this form of appendicitis with one death. One of the cases was operated on for him by Dr. Joseph Price. The results he has attained are no better than many other operators have had, and he attributes his success largely to the fact that, if possible, he operates during the quiescent stage, as originally suggested by Dr. Treves in 1886, when danger of sepsis has passed and inflammatory symptoms have disappeared. The danger is practically nothing if the surgery is clean.

Under the head of symptoms which determine the safety of postponing operative interference until the acute stage is passed, the author said when we remember that fully one-half of all the cases of appendicitis recover from the first attack; that spontaneous resolution takes place when peritonitis, exudation, and sometimes even suppuration, have been present; and that a large number of cases which recover without an operation in the primary attack remain well and have no recurrence of the disease, the question of when to operate or not to operate is a very nice, difficult, and always an important one.

Dr. McGuire agrees with Dr. Wyeth, that if we have a competent surgeon, one skilled in abdominal work, and the patient can be operated on within twelve hours of the first symptoms of the attack, 99 per cent. will be cured. But we frequently see the cases after these twelve hours have expired, and surgeons of experience in such work are not always within call. He is not in favor of an operation in all cases as soon as a positive diagnosis is made.

*Value and Limits of Saline Purgation.*—In an ordinary case, not a fulminating one, after a short time the pain abates, the fever lessens, the pulse gets slower, the abdomen less hard and tender, and all the urgent symptoms more or less subside—they do not disappear, but are less pronounced—and then there comes in a remedy which in his hands has been as certainly valuable for good as quinine in malaria or arsenic in neuralgia—that is, saline purgatives, given in frequent doses until the patient is freely and repeatedly purged.

*Relative Frequency of the Disease in the Two Sexes.*—From the reports of many surgeons we learn that appendicitis is much more common in males than females, the proportion stated being one to four. This has not, however, been the author's experience. In his cases for for the past year, it will be seen that thirteen were females and thirteen were males; and this has been about the proportion of all his cases, amounting now to 155, the cases being nearly equally divided between the two sexes.

With reference to the technique of operation, he has this to say: "In fulminating cases the abdomen should be opened by a long incision, the gangrenous appendix ligated and cut off, and the belly washed out with gallons of sterilized water. Six or eight strips of iodoform gauze should be carried through the incision to different parts of the cavity. The wound should not be sutured, but every facility afforded for easy drainage. If such a case is not seen until the bowels are distended with gas and the patient prostrated or collapsed, it is useless to operate. I have never seen a case of fulminating appendicitis without premonitory symptoms. A careful inquiry into the past history will always show former attacks, which possibly at the time were not recognized.

"In cases where there is a deep-seated localized abscess, it should be rendered accessible by a free abdominal incision. The abscess should be carefully walled off from adjacent tissues by pieces of gauze, and then opened. The pus should be sponged out and the appendix carefully searched for and removed. The abscess wall should be dissected out and the bowels examined to see that they are intact. It is impossible to treat such cases extraperitoneally; and when you have exposed the cavity to the danger of infection there is no more excuse for doing incomplete work than in other forms of abdominal surgery."

In cases where the abscess has approached the anterior abdominal wall and become adherent to the peritoneum, he simply opens and drains it, and makes no effort to extract the appendix if not loose, or to remove the wall. To endanger the infection of the general cavity by too prolonged an attempt to find the appendix is not good surgery.

In cases of chronic or relapsing appendicitis, Dr. McGuire does not advise an operation until the patient has had two attacks. Sometimes the inflammation which attends the first attack renders the patient free from a second attack because of some obliterating pathological change in the appendix. He makes one exception to this rule. If several weeks after the first attack he finds induration or tenderness, or both, over the region of cecum, he advises the patient to have the operation done at once. The operation should always be done between the attacks. In every case all adhesions should be freed, and any portion of the omentum in contact with the appendix, which is suspected of being contaminated, should be removed.

Dr. J. C. LeHardy, of Savannah, read a paper in which he outlined an act to create a Commissioner of Health and Drainage for the State of Georgia.

Dr. J. D. Chason, of Iron City, read a paper entitled "Puerperal Septicemia; its Cause and Treatment." The author regards a patient with this disease in a condition strikingly analogous to one bitten by a snake. A poison is introduced into the blood which causes minute changes in the tissues and also produces a decided shock to the nervous system. The patient may be prostrated by primary depression. If this can be averted here, as in snake bite, the patient will recover, unless the diffuse changes have too deeply involved vital organs. In this disease we must stop the generation and absorption of poison, and if this can be done we need not fear the slight damage done by the poison already absorbed.

The indications for the treatment were threefold : First, to stop the generation and absorption of the poison. Second, to neutralize the poison already absorbed by free stimulation. Third, to increase the action of the excretory organs with a view to the elimination of the poison already absorbed and to prevent further absorption.

Dr. R. R. Kime, of Atlanta, followed with a paper on "The Present Status of Puerperal Infection." The author said that the keystone to success in puerperal infection is elimination and drainage, counteracting absorbed toxins and germs, checking their further development, and sustaining the patient by such remedies and measures as will conserve the vital forces of patient the most. After more than ten years of practical demonstration of drainage, both uterine and alimentary in puerperal infection, the author is more fully convinced than ever of its utility. Not only does it lessen suffering, relieve more cases of puerperal infection, and save more lives than any other means now at command, but also prevents more pelvic complications, saves more uteri, tubes and ovaries from subsequent disease and leaves them in a condition to perform their normal functions, which cannot be said of any other plan of treatment known to the profession of to-day. All cases of puerperal infection should be fed systematically liquid, easily digested food, and where they fail to take sufficient nourishment some of the prepared foods should be given. Of medicinal remedies, quinine given in 5 to 10 grain doses, every four to six hours, acts well in many cases, tending to check germ development and inflammation, incidentally helping to control temperature without detriment to the patient. Strychnia combined with bitter tonics, antiseptics or digestants, given every four hours, with nourishment, is beneficial in most cases. In weak, debilitated cases some form of iron should be given. Hypnotics should be given if needed to procure rest or sleep. Stimulants in moderation, systematically given, will do good in very weak cases, yet strychnia, quinine and digitalis are more permanent and beneficial in their results.

Surgical measures are indicated when the infection becomes localized with pus formation, and some claim in general peritonitis. Pus accumulations in the broad ligaments, as a rule, should be drained through the vagina by Henrotin's method, whether they be due to cellulitis, peritubal inflammation or ruptured pus tubes. In pyosalpinx and ovarian abscess authorities differ as to whether the vaginal or abdominal route is best.

Hysterectomy is advocated in cases of thrombo-phlebitis, multiple abscesses in uterine wall, etc , but it is a very difficult matter to diagnose the condition requiring such a surgical procedure. Where the septic infection is so marked as to produce such serious lesions of the uterus, it has usually passed beyond the reach of hysterectomy before a differential diagnosis can be made demanding such an operation. While the infection is rapidly progressing the author doubts the utility of such a measure, but after the infection and inflammation have become localized in the uterus, tubes, or ovaries, then it is time to consider such operative measures as are best suited to the individual case.

Dr. J. L. Hiers, of Savannah, read a paper entitled "Some Interesting Ophthalmological Cases," in which he reported 87 cases of phlyctenular and corneal ulcers treated with tincture iodine, 74 cases requiring only one application, 9 cases two applications, 2 cases a third application, and 3 cases had to be cauterized by the actual cautery.

Dr. Geo. Horine, of Americus, read a paper on "Lupus of the Nose, its Medical and Surgical Treatment." The author reported three cases of this disease which were successfully treated by the use of pyoktanin.

Dr. J. M. Crawford, of Atlanta, reported a successful rhinoplastic operation for loss of the nose. He also reported a case in which he operated for removal of the drum membrane and necrosed bones in chronic suppurative aural catarrh.

The following officers were elected:

President—Dr. J. B. Morgan, of Augusta.

First Vice-President—Dr. L. G. Hardman, of Harmony Grove.

Second Vice-President—Dr. J. L. Hiers, of Savannah.

Next place of meeting, Cumberland Island; time, third Wednesday in April, 1898.

